# Supplementary material for: Polyketide synthase gene clusters in the ericoid mycorrhizal fungus Oidiodendron maius: genome-wide prediction, expression profiling, and functional characterization of knockout mutants
Source: Front Plant Sci. 2026 Sep 16;17:1924430. doi: 10.3389/fpls.2026.1924430 (PMC13623903; doi:10.3389/fpls.2026.1924430)
Supplement: Supplementary file 1 [file SupplementaryFile1.zip › Supplementary Materials/Supplementary Folder 1.zip!/Oidma1_AssemblyScaffolds_Repeatmasked/knownclusterblast/cluster49/jgi.p_Oidma1_45885_mibig_hits.html]

| MIBiG Protein | Description | MIBiG Cluster | MiBiG Product | % ID | % Coverage | BLAST Score | E-value |
| --- | --- | --- | --- | --- | --- | --- | --- |
| AGN71623.1 | salicylate\_hydroxylase | BGC0000027 | Azaphilone\_biosynthetic\_gene\_cluster | 50 | 97.9910714286 | 456 | 4.4e-128 |
| EHA28235.1 | hypothetical\_protein | BGC0001143 | Azanigerone\_biosynthetic\_gene\_cluster | 45 | 97.5446428571 | 388 | 8.5e-108 |
| EED18000.1 | monooxygenase,\_putative | BGC0000154 | Stipitatic\_acid\_biosynthetic\_gene\_cluster | 42 | 98.2142857143 | 360 | 3.3e-99 |
| EAQ86391.1 | hypothetical\_protein | BGC0001405 | Chaetoviridin\_/\_chaetomugilin\_biosynthetic\_gene\_cluster | 41 | 96.875 | 351 | 2e-96 |
| EAA65601.1 | hypothetical\_protein | BGC0000022 | Asperfuranone\_biosynthetic\_gene\_cluster | 40 | 99.3303571429 | 325 | 1.2e-88 |
| CAP95403.1 |  | BGC0001404 | Sorbicillin\_biosynthetic\_gene\_cluster | 39 | 99.1071428571 | 324 | 2.6e-88 |
| EAU32816.1 | conserved\_hypothetical\_protein | BGC0000160 | Terreic\_acid\_biosynthetic\_gene\_cluster | 37 | 95.7589285714 | 225 | 1.6e-58 |
| AME18017.1 | epoxidase | BGC0001378 | Tiancimycin\_biosynthetic\_gene\_cluster | 27 | 88.8392857143 | 104 | 2.5e-22 |
| AAP85357.1 | putative\_monooxygenase | BGC0000233 | Hedamycin\_biosynthetic\_gene\_cluster | 26 | 87.9464285714 | 84 | 4.5e-16 |
| AEE65479.1 | oxidoreductase | BGC0000223 | Fluostatin\_biosynthetic\_gene\_cluster | 26 | 90.625 | 81 | 2.9e-15 |
| AAS92536.1 | HDX1 | BGC0001044 | Sirodesmin\_biosynthetic\_gene\_cluster | 24 | 93.75 | 77 | 3.2e-14 |
| CRG85571.1 | hypothetical\_protein | BGC0001402 | Cyclochlorotine\_biosynthetic\_gene\_cluster | 25 | 83.4821428571 | 66 | 9.7e-11 |
| BAA84784.1 | Vio\_C | BGC0000828 | Violacein\_biosynthetic\_gene\_cluster | 25 | 48.4375 | 50 | 4.2e-06 |
| AAD51810.1 | monooxygenase\_VioC | BGC0000829 | Violacein\_biosynthetic\_gene\_cluster | 25 | 48.4375 | 50 | 4.2e-06 |
